# “Digital Dividend” or “Digital Divide”: What Role Does the Internet Play in the Health Inequalities among Chinese Residents?

**DOI:** 10.3390/ijerph192215162

**Published:** 2022-11-17

**Authors:** Dongling Zhang, Guoqing Zhang, Yuxin Jiao, Yanyan Wang, Pengnian Wang

**Affiliations:** School of Economics, Qingdao University, Qingdao 266061, China

**Keywords:** health inequality, Internet usage, income inequality, mental health, health consciousness, Internet access discrepancy, quantile regression

## Abstract

With the vigorous development of the medical industry in China, residents’ health has been significantly improved. However, along with the income gap, urban–rural gap, and healthcare resource gap caused by economic development, health inequality has become a fundamental barrier to the promotion of residents’ health. The popularity of the Internet has helped close the gap to some extent, but it also has drawbacks. Using data from the China Family Panel Studies (CFPS) from 2014 to 2018, we evaluated the effects of Internet usage on health disparities among residents using fixed effect models, mediation effect models, and other methodologies. The findings indicate that Internet usage can help to minimize health inequality since it lowers income inequality, promotes health consciousness, and reduces depression. Furthermore, Internet usage plays a greater role on the health improvement of the middle-aged, the elderly, urban residents, and females. Although the Internet has brought “digital dividends” in general, the Internet usage rates among different groups also reveal that there is a clear “digital gap” among rural residents, elderly groups, and low-income groups. These results have significant implications for promoting healthcare equality.

## 1. Introduction

Over the past 40 years, China has experienced rapid economic growth and a dramatic increase in economic power [1], leaping to become the second-largest economy in the world. As a result of this phenomenal economic growth, millions of Chinese people have been lifted out of poverty, making China a model for poor countries. While for many, economic growth has brought prosperity, there is a widening gap between those who have benefited from economic development and those who have been left behind. Large development gaps exist between regions, urban and rural areas, and social groups. The creation of health disparities can destabilize societies [2]. Over the past few decades, there have been significant differences in health care facilities, a polarized health care system, and an economic dichotomy between urban and rural areas, which have continued to widen the gap in health status between urban and rural areas [3].

To illustrate the extent of health care disparities in China, we examined statistical reports published by the China Health and Wellness Commission in 2021. Child mortality is an outpost event to assess the quality of health care and is a common indicator to assess health status. The statistical report states that the under-five mortality rate in China in 2021 was 7.1 per 1000 children, including 4.1 per 1000 in urban areas and 8.5 per 1000 in rural areas. Statistics show that child mortality rates are much higher in underdeveloped areas of China than in developed areas. China’s child mortality rate has been going down for decades, which means that China’s health care is always improving. However, there is always a difference between urban and rural areas and between regions, and China has both backwardness and prosperity [4].

As part of its efforts to reduce social disparities, China is working to narrow its health inequalities. The Chinese government is not only working to advance the nation’s common prosperity, but also committed to equalizing public services. As one of its priorities, China has undertaken a comprehensive health care reform to ensure equitable and affordable health care for all citizens [5]. Among the specific approaches are the equalization of urban and rural health care services and graded care, and these reforms have alleviated health inequalities among Chinese residents [6,7]. However, with the spread of Internet technology and rapid urbanization in China, regional disparities in China have shown rapid changes [8]. Specifically, the large-scale use of Internet technology has not only played an important role in promoting regional economic growth by increasing efficiency [9,10] and innovation [11], but it has also effectively used the Internet as a vehicle to remove geographical barriers to economic interaction and reduce transaction costs, powered by ICT. Along with the rapid expansion of the Internet, society is undergoing profound changes as the network and society are fully integrated [12]. Therefore, in the field of regional economics, the Internet is considered a key factor affecting disparities [13]. Some scholars believe that the continued penetration of the Internet can connect otherwise isolated regions to each other. Additionally, the Internet can enable some resource-scarce regions to gain access to more markets and opportunities. This suggests that the Internet has an important role in reducing regional economic disparities [14].

In 2013, China’s State Council released the “Broadband China” strategy, which will be implemented in 120 cities in batches in 2014, 2015, and 2016. The strategy aims to increase Internet penetration, improve Internet infrastructure coverage, increase network speed, and promote industrial upgrading and innovation. According to the Digital Economy Report 2021 published by the United Nations Conference on Trade and Development, the United States and China are the main driving forces as well as the main beneficiaries of the digital economy. These two countries have half of the world’s hyperscale data centers and lead the world in 5G penetration. According to a report released by the China Internet Network Information Center, as of June 2022, the number of China’s Internet users was 1.051 billion, and the Internet penetration rate reached 74.4%, and the number of China’s Internet users continues to grow steadily. The above figures illustrate the rapid development of the Internet in China, which corresponds to the impact of the Internet on all aspects of Chinese residents’ lives.

However, most of the research on the Internet’s impact on inequality has focused on the macroeconomic level, and research focusing on the Internet and health inequality remains scarce. One reason such research is necessary is that disparities in access may be due to non-economic factors. Thus, a reduction in disparities at the economic level may not represent a reduction in health disparities. As the Internet is currently widely used by Chinese residents, on the one hand, people use it for leisure and entertainment, knowledge acquisition, and even work and entrepreneurship, which contributes to residents’ mental health and health literacy and can improve their income. On the other hand, the Internet has also caused a series of problems, such as the existence of uneven information on the Internet, which makes people easily addicted to it. Moreover, the Internet requires hardware and software support, which means that there are barriers to Internet use, which may generate differences in Internet access. In summary, the role of the Internet in health inequities may be both “dividend” and “divide”. What exactly is the role of the Internet in health inequalities? How does the Internet affect health inequalities? The purpose of this paper is to provide an in-depth analysis of how the Internet affects health inequalities in the macro context of the rapid spread of the Internet and the ongoing health inequalities based on data from the China Family Panel Studies (CFPS). We also explore through which channels the Internet acts on health inequalities and what role each of these channels plays. In addition, we examine the role played by the Internet in populations with different characteristics. The analysis of these issues has important implications for health care access equity, being able to severing as not only a theoretical complement to the Internet’s contribution to health inequality, but also a basis for decision-making on Internet development and the reduction of social health inequality.

## 2. Literature Review and Research Hypothesis

### 2.1. Literature Review

The research in this paper builds on the literature in two categories, the first being about measures and determinants of health inequality. The second category is about the impact of the Internet on health and health inequality.

When we discuss the measures of health inequality, we first need to clarify the two types of health inequality definitions. Health inequality in a narrow sense is the difference in the distribution of health levels among different populations, i.e., inequality in outcomes, while health inequality in a broad sense includes both differences in health status and differences in access to and conditions for good health [15,16,17]. Based on different perspectives, research methods on health inequalities are also broadly divided into two categories. The index method and the regression method are the two dominant methods for measuring health inequalities. The exponential method can be classified into two main types. One is the measurement of the concentration index of health, such as the calculation and decomposition of the Gini coefficient, generalized entropy index, Atkinson index, Erreygers index, and Kobus–Milos index [18,19,20]; the other is the calculation of the relative deprivation index of health [21] and the multidimensional health deprivation index [22]. The index method is able to measure health outcome inequality and can reveal the mechanism of influence of factors such as income disparity on health inequality through factor decomposition. The regression approach is to explore whether the effect of limited factors on health inequality is significant through modeling regression analysis and can be used to test the effect of differences in access to good health on health inequality [23,24]. Based on data availability and ease of measurement, we prefer to use differences in health status as a proxy for health inequality. We also use the relative deprivation index of health to measure health inequality so that regressions and calculations in microdata can be done more easily.

The determinants of health inequality can be broadly classified into two categories. The first category is personal factors, such as an individual’s age, education, gender, race, employment status, and income level [25]. In addition, childhood encounters can have long-term effects, such as poverty [26], war [27], famine [28], influenza [29], weather changes [30], economic recession [31], and natural disasters [32], which can negatively affect nutritional supply and thus have lasting adverse effects on physical and mental health in adulthood, increasing the incidence of diseases such as heart disease, diabetes, and depression. These personal factors may also have an impact through indirect pathways. For example, an increase in personal income can increase health inputs, such as the purchases of health insurance or health care facilities [33], and the efficiency of the use of medical and health services [34], thus maintaining individual health levels. People with higher levels of education tend to have more health knowledge and are more likely to discipline their own behavior and adopt good lifestyle habits, such as reducing smoking and alcohol consumption and increasing exercise activities to reduce the decline of health capital [35]. A higher level of education also means a higher level of understanding, which helps people develop an objective view of their own health [36]. It also makes it easier to control negative emotions and keep their mental health in good shape [37].

The second category is social factors, where socioeconomic factors are considered fundamental factors affecting health inequalities [38]. The impact of different socioeconomic conditions on health inequalities has been demonstrated, and it has been found that increased income disparity leads to unhealthy habits, lack of material resources, and shortage of social capital in low-income groups [39,40], and negative emotions such as depression and loneliness in low-income groups also increase with age [41,42]. Thus, the health of the rich compared to the poor is expanding, increasing pro-rich health inequalities [43]. People with higher socioeconomic status have greater access to and utilization of health care, increasing health inequality [44,45]. The level of education indirectly affects the level of health inequality, including through lifestyle changes [46,47]. Social public services can reduce health inequalities, and Zheng et al. [48] found that the construction of community sports facilities helped to improve the mobility of the elderly and promote social interaction among them, thus playing a role in bridging health inequalities. Differential exposure levels to environmental pollution are also an important source of health inequality, and groups with lower socioeconomic status are more likely to be exposed to environmental pollution and bear higher health risks [49]. Based on this, population migration becomes a way to avoid health risks caused by environmental pollution, but there are health selection mechanisms for migration that widen health inequality between inflow and outflow [50].

The Internet has an impact on health, and that impact continues changing as the Internet evolves. In recent years, as the Internet continues to embed itself into social life, information technology has been reshaping the social fabric by influencing patterns of information flow and interaction between individuals. Internet use does not directly affect health, but rather has an impact on health and health inequalities through indirect channels. There are two distinct viewpoints. One view is the health promotion theory, which suggests that Internet use and the use of digital information technologies bring a “digital dividend” and that the Internet can promote the mental health and physical health of users and bridge health inequality [51]. First, the Internet can promote users’ social participation, social activities, and recreation, thus promoting mental health [52]. Second, as an important medium for disseminating information, the Internet plays an important role in disseminating health information, and users can use the Internet to find health knowledge, enhance prevention and care, and improve lifestyle habits, thus improving physical health [53]. As for health inequalities, some scholars have pointed out that the Internet as an important information carrier regulates the impact of socioeconomic conditions on health inequalities, and the popularity of the Internet has to some extent bridged the access divide, and the increase in health information on the Internet has changed the distribution pattern of health information [54], thus promoting the leveling of information distribution and the networking of information flow, satisfying the health needs of otherwise information-disadvantaged groups and helping to reduce health inequalities across different classes [55]. Another view is that people may not be able to adapt to the Internet in the right way and, thus, the health risks [56], such as overuse of the Internet and dependence on online social media, can have a negative impact on the health of users [57].

Based on existing research, scholars are still divided on how the Internet affects health inequality, with most of them affirming the role of the Internet in reducing economic disparities and creating equal access to health care resources. However, there is still a lack of evidence on the role of the Internet in health inequalities and its mechanisms, on the one hand, and a lack of micro-level evidence on the other. In addition, we would like to confirm whether a “digital divide” still exists in modern society, i.e., significant differences in access and use of the Internet across social classes, which implies a new social inequality. As China’s economy grows, equity in access to health care resources and health equity among the population will be important factors in promoting social equity in China. Therefore, researching how the Internet contributes to health disparities is a crucial subject. For these reasons, we analyzed the routes and variations in the impacts of the Internet on health disparities using data from the China Family Panel Studies. Finally, we discussed whether the Internet “divide” exists.

### 2.2. Research Hypothesis

First, the development of the Internet has profoundly changed the traditional economy, and residents can enjoy lower transaction costs and a larger market size through the Internet. An unobstructed resource network can boost residents’ economic income, and the Internet compensates for income inequality due to resource scarcity. Residents enjoy a more equitable environment for entrepreneurship and innovation due to the Internet. In turn, the decrease in income inequality means the equalization of people’s nutritional conditions, living conditions, and affordable medical expenses. That is, if the Internet can reduce income inequality, a decline in income inequality can reduce health inequality. Second, the use of interconnection has a huge impact on the psychology of the population due to the huge amount of entertainment information carried by the Internet, which allows the population to enjoy leisure well. In addition, the Internet makes it easier to communicate with relatives, which facilitates the maintenance of kinship among residents. For individuals who are psychologically depressed, the Internet helps improve their health status, suggesting that the Internet may reduce health inequalities by reducing depression. Finally, the Internet has shared more health perspectives and knowledge. Modern lifestyles have made people more concerned about their bodies and health. Therefore, when the Internet can effectively deliver health knowledge, people’s health consciousness will increase; in other words, people will care more about their bodies. This can encourage people to increase the frequency of physical exercise and prevent chronic diseases as well as obesity. Therefore, the Internet may reduce health inequality by raising the health consciousness of the population. Based on the above analysis, we derive the following hypotheses:

**Hypothesis 1 (H1).**  *The Internet can reduce health inequality*.

**Hypothesis 2 (H2).**  *The Internet reduces health inequality by reducing income inequality.*

**Hypothesis 3 (H3).**  *The Internet reduces health inequality by reducing depression.*

**Hypothesis 4 (H4).**  *The Internet reduces health inequality by raising health consciousness.*

## 3. Materials and Methods

### 3.1. Data Source and Sample Selection

The data used in this research were obtained from China Family Panel Studies (CFPS). The CFPS sample covered 25 provinces, and the survey subjects included all household members in the sample households. In view of the purpose of the research and data availability, the panel data from 2014 to 2018 were selected and processed as samples of this research. First, we matched and merged the adult database in the CFPS database with the family economic database. Second, we removed samples with the main variables missing and processed some missing values that could be filled in, such as age, gender, and household registration type. To further reduce the regression bias errors, we added macroscopic variables obtained from the China Statistical Yearbook and the Wind database to the models and merged them with the CFPS data by province. Finally, we combined the data into the balanced panel data and obtained 51,375 observations.

### 3.2. Variables Selection

#### 3.2.1. Explained Variable

We used health self-assessments to measure health inequality. The advantage of this approach is that we can directly conduct research at the microlevel. According to relative deprivation theory, if an individual has a lower level of health within a cohort, the bigger the health disadvantage and the higher the degree of relative health deprivation endured, that is, the higher the level of health inequality. According to the definition of relative deprivation index (RD) proposed by Kakwani [21], assuming that *Y* represents a reference group with a sample size of *n*, we rank the self-rated health levels of the samples in the group to obtain vector $Y=\left(y_{1},y_{2},y_{3},\ldots ,y_{n}\right)$ as the overall self-rated health distribution. Notably, where $y_{1}\leq y_{2}\leq y_{3}\leq \ldots \leq y_{n}$. So, if we compare the health of the *i*th resident to that of the *j*th resident, we can express the relative health deprivation index $RD\left(y_{j},Y_{i}\right)$ of the *i*th resident as follows:

(1) $$RD\left(y_{j},y_{i}\right)=\{\begin{array}{c} y_{j}-y_{i}\;\;\;\;if\;\;\;\;y_{j}>y_{i}\\ 0\;\;\;\;\;\;\;\;\;\;\;\;\;\;if\;\;\;\;\;y_{j}\leq y_{i} \end{array}$$

On the basis of the above equation, the average relative deprivation $RD\left(y,y_{i}\right)$ suffered by the *i*th resident is as follows:

(2) $$RD\left(y,y_{i}\right)=\frac{1}{n\mu_{Y}}\left(n_{y_{i}}^{+}\times \mu_{y_{i}}^{+}-n_{y_{i}}^{+}\times x_{i}\right)$$

where $\mu_{Y}$ is the mean value of self-rated health of all *n* samples in the reference group *Y*, $n_{y_{i}}^{+}$ is the number of samples with self-rated health level over $y_{i}$ samples in the reference group *Y*, and $\mu_{y_{i}}^{+}$ is the mean value of the samples with self-rated health level over $y_{i}$ in the reference group *Y*. The average relative deprivation suffered by the *i*th resident is used as a proxy variable for health inequality in this research.

#### 3.2.2. Explanatory Variable

The primary explanatory variable in this research is Internet use, which is a dummy variable with a value of 1 when residents use the Internet and 0 otherwise. We identified whether residents use the Internet based on two questions in the CFPS questionnaires. These two questions are “Do you access the Internet on a mobile device?” and “Do you access the Internet on a computer?” Residents are deemed Internet users if they answer “yes” to either question. Notably, in the 2014 data, we estimated the Internet use based only on the response to the question “Do you use the Internet?”.

#### 3.2.3. Mediating Variables

In this research, there are three mediating variables: income inequality, depressive mood, and physical activity frequency. These three variables are very reflective of the study’s action mechanisms. First, income inequality creates inequalities in nutrition and living circumstances, as well as differences in access to health care resources, which have a great effect on health inequality. Second, differences in mental health levels are an important factor in health inequality, and the severity of depression can also have an effect on health inequality. Third, the frequency of physical activity reflects the residents’ health consciousness, and the enhancement of health consciousness will have a mitigating influence on health inequality. The three mediation settings in this paper are as follows:

We use a measure similar to that of health inequality to figure out how unequal income is. However, unlike health inequality, we standardize income inequality after the measure because income is numerically larger. This makes it easier to express the regression coefficients without affecting how well the regression works. We used the depression scale CES-D score to reflect the severity of depressive mood. It is worth noting that the CFPS database used a 6-item CES-D scale in 2014, a 20-item full CES-D scale in 2016, and an 8-item CES-D scale in 2018. In order to maintain the comparability of the data, we used a 6-question scale to calculate the score in 2014–2018. Residents responded to the questions with “nearly never”, “occasionally”, “often”, and “most of the time”, which were split into 0–3 points, and the scores for each question were then added together to determine the degree of resident depression. The frequency of physical activity was measured using the question “How often did you exercise in the past week” from the CFPS questionnaire, with values ranging from 0 to 50.

#### 3.2.4. Control Variable

On the basis of existing research, this paper selects age, gender, whether urban population, per capita household net income, family size, marriage, and household registration type as control variables at the resident level. In order to further control the model bias, we added 5 macroeconomic variables to reflect the development differences in different provinces, which are the level of economic development, urbanization rate, government size, fiscal expenditure, and healthcare resources.

Resident-level control variables are directly given by the questionnaire in CFPS, in which per capita household net income has been adjusted to comparable data. Among the control variables at the macro level, the level of economic development is measured by per capita GDP; the urbanization rate is measured by the ratio of urban population to the total population; the scale of government is measured by the ratio of local fiscal revenue to GDP; fiscal expenditure is measured using the general fiscal budget expenditure; and the measurement of healthcare resources uses the ratio of the number of medical institutions to the resident population. We take the logarithm of per capita household net income, economic development level, and fiscal expenditure in order to mitigate possible multicollinearity and heteroskedasticity.

### 3.3. Research Methods

#### 3.3.1. Basic Model

Considering the model’s rigor, we adopted a two-way fixed effects model since there are evident individual and temporal features among the study’s resident people. After introducing individual fixed effects and temporal fixed effects to the model, the model was configured as follows:

(3) $$HI_{it}=\alpha +\beta Int_{it}+\gamma Z_{it}^{\prime }+u_{i}+\lambda_{t}+\varepsilon_{it}$$

where $HI_{it}$ denotes the degree of health inequality, $Int_{it}$ denotes whether or not to use the Internet, $Z_{it}^{\prime }$ denotes all control variables in the model, $u_{i}$ denotes province fixed effects, $\lambda_{t}$ denotes time fixed effects, and $\varepsilon_{it}$ is the random disturbance term.

#### 3.3.2. Mediating Effect Model

We also evaluated whether economic inequality, depressive mood, and physical activity frequency served as mediation factors for the impact of Internet usage on health inequality. Referring to previous studies [58,59], we performed stepwise regression to examine the mediating effects. The design was as follows:

(4) $$HI_{it}=\alpha +\beta_{1}Int_{it}+\gamma Z_{it}^{\prime }+u_{i}+\lambda_{t}+\varepsilon_{it}$$

(5) $$M_{it}=\alpha +\beta_{2}Int_{it}+\gamma Z_{it}^{\prime }+u_{i}+\lambda_{t}+\varepsilon_{it}$$

(6) $$HI_{it}=\alpha +\beta_{3}Int_{it}+\beta_{4}M_{it}+\gamma Z_{it}^{\prime }+u_{i}+\lambda_{t}+\varepsilon_{it}$$

where $HI_{it}$ is health inequality, $Int_{it}$ is whether or not to use the Internet, $M_{it}$ is the mediating variable, $Z_{it}^{\prime }$ is all control variables, and $u_{i}$, $\lambda_{t}$, and $\varepsilon_{it}$ represent province fixed effects, time fixed effects, and random disturbance terms, respectively. Significant coefficients $\beta_{1}$, $\beta_{2}$, and $\beta_{4}$ indicate the presence of mediating effects, while insignificant coefficient $\beta_{3}$ indicates full mediation.

#### 3.3.3. Treatment Effect Model

Since the decision whether or not to use the Internet is decision-generated and not derived from a completely exogenous random grouping, individuals with high levels of household income may be more likely to access the Internet. Moreover, the high level of economic development and good Internet infrastructure in residents’ areas may be more likely to lead residents to use the Internet, and there may be some selection bias. To alleviate the decision selection bias, a treatment effect model is constructed by introducing the inverse Mills ratio for modeling whether to use the Internet, where the selection equation is:

(7) $$D^{*}=\alpha +\delta Z_{it}^{\prime }+\varepsilon_{it}$$

where the individual uses the Internet when $D^{*}\geq \;0$. $Z_{it}^{\prime }$ denotes explanatory variables affecting whether the individual uses the Internet, and we used the number of members of the same household with Internet access and the topographic relief of the province where the resident is located.

#### 3.3.4. Quantile Regression

We want to apply quantile regression to estimate the impact of Internet use on residents with varying degrees of health deprivation in order to distinguish between the impact of Internet usage on residents with severe and moderate health deprivation. We used the quantile regression [60,61], which not only offers the regression coefficients at each quantile for this investigation, but also makes this study more resistant to extreme results. This approach’s model was established as follows:

(8) $$y_{\tau }\left(x_{i}\right)=x_{i}^{\prime }\beta_{\tau }$$

where *τ* denotes the quantile and $\beta_{\tau }$ denotes the coefficient on the *τ*th quantile. The estimation of $\beta_{\tau }$ is expressed using the following method:

(9) $$\underset{\beta_{\tau }}{\min}\sum_{i:y_{i}\gg x_{i}^{\prime }\beta_{\tau }}^{n}\tau \left|y_{i}-x_{i}^{\prime }\beta_{\tau }\right|+\sum_{i:y_{i}\gg x_{i}^{\prime }\beta_{\tau }}^{n}\left(1-\tau \right)\left|y_{i}-x_{i}^{\prime }\beta_{\tau }\right|$$

## 4. Results

### 4.1. Descriptive Statistics

Table 1 provides descriptive data for the major factors. Table 1’s descriptive data reveals that China’s average Internet use is 35.1%. Taking into account the survey period of 2014 to 2018 and the fact that the sample includes both rural and urban populations from all provinces, the Internet penetration rate in China is not low. However, due to China’s enormous population, there is still a sizeable portion of the population that does not use the Internet. When comparing the means of Internet users to those of non-Internet users, it is evident that Internet users had lower levels of HI and greater levels of health. In addition, the Internet-using group has lower income inequality, CES-D scores, and frequency of physical activity. In terms of the individual characteristics of both groups, Internet users are younger, more likely to reside in urban areas, more likely to be male, and have a greater number of single individuals. In terms of household characteristics, Internet users had a greater per capita household income and a larger family size. In terms of macroeconomic factors, the majority of economic indicators in locations where Internet users reside are likewise greater than those of non-Internet users. All of the aforementioned mean differences passed the *t*-test at the 1% significance level (except for the 5% significance of family size), indicating that this difference is statistically significant. We added personality traits, household characteristics, and regional economic factors to the model to minimize regression bias. To avoid being swayed by these biases, the next section gives a more in-depth look at how the Internet works and how it does it.

### 4.2. Benchmark Regression Results

To test the robustness of the regression findings, we ran the regressions with a stepwise inclusion of control variables, as shown in Table 2. We use a stepwise addition of control variables to confirm the robustness of the regression models. When we first restrict the model to the primary explanatory variables, the regression coefficient is noticeably negative. Then, we begin to include control variables for individual characteristics, household characteristics, and macroeconomic factors. Finally, we added individual fixed effects to the model. The regression coefficient for the Internet is −0.920 when we do not include control variables, and it is significant at the 1% level. The regression coefficient changes to −0.193 when we include the control variable for individual characteristics, and it stays significant at the 1% level after including the control variable for household characteristics. The coefficient becomes −0.142 when the macroeconomic factors are added on. When fixed factors are added to the model, the regression coefficient finally settles at −0.187, and it is still significant at the 1% level. This suggests that utilizing the Internet may dramatically decrease HI and improve the degree of health deprivation, and that this conclusion is robust even when control factors are gradually included. Therefore, Assumption 1 holds. From the control variable regression findings, we may derive the following conclusions: Increasing age exacerbates HI; HI is higher in female and rural groups; raising income levels, increasing the number of family members, and improving regional economic levels may reduce HI.

### 4.3. Robustness Test

#### 4.3.1. Change the Variables

We used the method of changing variables in order to test the robustness of the conclusions drawn and to make sure that the conclusions obtained were not due to chance. We replaced Int with time spent on the Internet and reran the regression. The results obtained are shown in model (6) in Table 3. According to the results obtained from model (6), time spent online has a significant negative effect on health inequality. This shows that more time spent on the Internet reduces health inequality. This is in line with the main findings, and the conclusions reached are strong.

#### 4.3.2. Endogenous Test

We are concerned that the model may have endogeneity issues, for example, groups with poorer health may have more difficulty accessing the network. Therefore, we used an instrumental variable approach to mitigate the endogeneity of the model. We used the number of members of the same household with Internet access (except for the residents themselves) as an instrumental variable. This is due to the fact that residents themselves are more likely to use the Internet if the number of family members using the Internet is higher. Additionally, since the decision of whether other household members are online or not is less related to themselves, the number of Internet users in a home is a more desirable instrumental variable. We used the number of Internet members as an instrumental variable for Int and regressed the model using the instrumental variables method, and the obtained results are shown in model (7) in Table 3. The coefficient of Int in model (7) remains significantly negative at the 1% level with a coefficient of −0.975. This shows that even when the endogeneity in the model is taken into account, the Internet still has a negative effect on health inequality.

#### 4.3.3. Selection Error Test

Since whether or not to use the Internet is a decision-generating and not derived from a completely exogenous random grouping, individuals with higher levels of household income may be more likely to have access to the Internet. In addition, regions with high levels of economic development and a good Internet building base may be more likely to have residents using the Internet, which in turn may lead to biased estimation results. To mitigate the potential selection bias of the model, the Int variable is modeled and the treatment effect model is constructed by introducing the inverse Mills ratio, where the explanatory variables of the selection equation maintain the number of Internet users in the same household and are supplemented by the province’s topographic relief where the resident resides. The regression results of the treatment effect model are shown in model (8) of Table 3. In model (8), the regression coefficient corrected by the inverse Mills ratio is still significant at the 1% level, with a regression coefficient of −0.922. This shows that the original conclusion does not change when the model’s selection bias is taken into account.

#### 4.3.4. Propensity Score Matching

As was previously indicated, the economic situations and household status of various inhabitants may introduce bias into the regression findings. Due to the fact that the sample covers 51,375 observations, there are vast disparities in age, wealth, etc., among the people. We included control variables to lessen the impact of this difference on the regression findings, but since this bias may not be linear, and the linear regression technique may conceal this bias, resulting in bias in functional form. Consequently, we used propensity score matching (PSM) to match each resident with people who are similar to them. We computed the propensity score based on all control factors and used the proximity matching approach to match the Internet population one-by-one with the five observations most similar to their circumstances; the resultant treatment impact is presented in model (9) in Table 3. In model (9), the PSM technique yields significant findings at the 1% level, with a coefficient of −0.920. This shows that the results hold up even when individual bias and functional form bias are taken into account.

### 4.4. Test of Mediating Effect

We tested for mediating effects in order to further investigate how Internet use acts on health inequalities. As mentioned earlier, Internet use can affect inequality in income, depressed mood, and health consciousness of the population. We used models of mediating effects to see if this effect was real, and the results are shown in Table 4.

Our test for the effect of income inequality is reflected in models (10) and (11). In model (10), we can see that the use of the Internet reduces the level of income inequality, which is significant at the 1% level with a coefficient of −0.020, while the effect of the level of income inequality on HI is positive, which is significant at the 1% level with a coefficient of 0.698. That is, the use of the Internet reduces the health disparity of the population by reducing the income disparity of the population, and the Internet has a dual effect of reducing income and health disparities. Model (12) shows that the use of the Internet reduces the depression of the population, and this finding is significant at the 5% level with a coefficient of −0.078, while model (13) demonstrates that the rise in depression of the population increases the HI status, and this finding is significant at the 1% level. Thus, the internet can alleviate the generation of depression in residents, which comes in many ways, and it has been suggested that the internet promotes interpersonal and social relationships [62], while this study further found that this effect can alleviate HI. Therefore, the internet may be more important for the unhealthy population, which we will discuss in more depth in the heterogeneity analysis. Using the same approach, we can find from models (14) and (15) that the Internet can enhance the frequency of physical activity among the population and promote the reduction of health disparities. This shows a positive effect of the Internet on health, which may arise from the widespread dissemination of health knowledge and the improvement of residents’ health literacy. This makes residents pay more attention to maintaining and enhancing their health, thus increasing their frequency of exercise and thus reducing HI. Therefore, hypotheses 2, 3, and 4 hold.

Through the above analysis, we found that the Internet can act on HI through Ini, Dep, and Exe. This indicates that the Internet plays a wide range of roles in the lives of residents, and the Internet can generate economic benefits, psychological comfort, and health literacy for users. Overall, the role of the Internet is positive, and the Internet effectively reduces HI in a variety of ways.

### 4.5. Heterogeneity Analysis

#### 4.5.1. Heterogeneity Analysis of Different Groups

**Figure 1:**
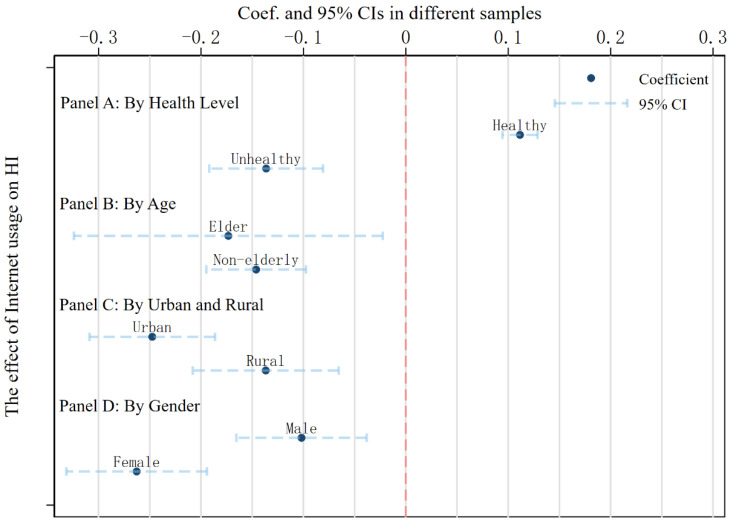
Heterogeneity test results.

In the above study, we treated all samples as homogeneous. However, the effect of the Internet on HI may be heterogeneous. To further investigate the heterogeneous effect of Internet use on HI, we used grouped regression to investigate the heterogeneity of the effect of the Internet on HI. We grouped the samples according to their characteristics: (1) We divided the full sample into healthy and unhealthy groups. We classified observations with health self-assessment value above the mean value as the healthy group and the remaining observations as the unhealthy group; (2) we divided the samples into older and non-older groups. According to the usual practice, residents who are older than 60 years of age are classified as the older group and those who are under or equal to the age of 60 are classified as the non-older group; (3) we classify residents into urban groups and rural groups according to the individual’s place of residence; and (4) we classify the sample into male group and female group according to gender. The regression results are shown in Figure 1.

Panel A of Figure 1 reveals that the internet regression coefficient for the healthy population is 0.111 and is statistically significant. In the unhealthy population, a negative internet regression coefficient of −0.136 is observed. This data suggests that Internet use among the healthy population enhances HI, but Internet usage among the unwell population has the reverse impact. A probable explanation for this phenomenon is that the healthy population is more prone to disregard the harmful effects of the Internet on their health; as a result, they may have a more serious Internet addiction that has negative consequences. Due to the prevalence of health issues, the Internet is more likely to have a beneficial impact on the unhealthy population. In panel B, the regression by age group demonstrated that the Internet had a bigger impact on the elderly. The regression coefficient for Internet use was −0.173 in the elderly population and −0.146 in the non-elderly population. This conclusion demonstrates that the Internet has a greater marginal impact on the elderly than on the younger population. In terms of health, elderly individuals need the Internet more than younger ones.

In panel C, we find that the regression coefficient of the internet in urban areas is −0.248 and is significant at the 1% level, while the regression coefficient of the internet in rural areas is −0.137, again significant at the 1% level. From the regression coefficient, the marginal effect of the Internet is greater in urban areas than in rural areas, which may be due to the fact that urban areas rely more on the Internet for information transmission. Compared to rural areas, urban dwellers are more isolated, families are more nuclearized, and it is more common for extended families to be split into smaller households to live. As a result, urban areas rely more on the Internet for communication to reduce depression and increase health consciousness among residents. Similarly, we compare the male group with the female group, and panel D shows the regression results for the male group with a regression coefficient of −0.102, which corresponds to a regression coefficient of −0.263 in panel D. We find that the marginal effect of the Internet is stronger in the female group. We believe a possible reason for this is that the female group in our sample is more depressed and, therefore, the marginal effect from the Internet is stronger.

#### 4.5.2. Quantile Regression

**Figure 2:**
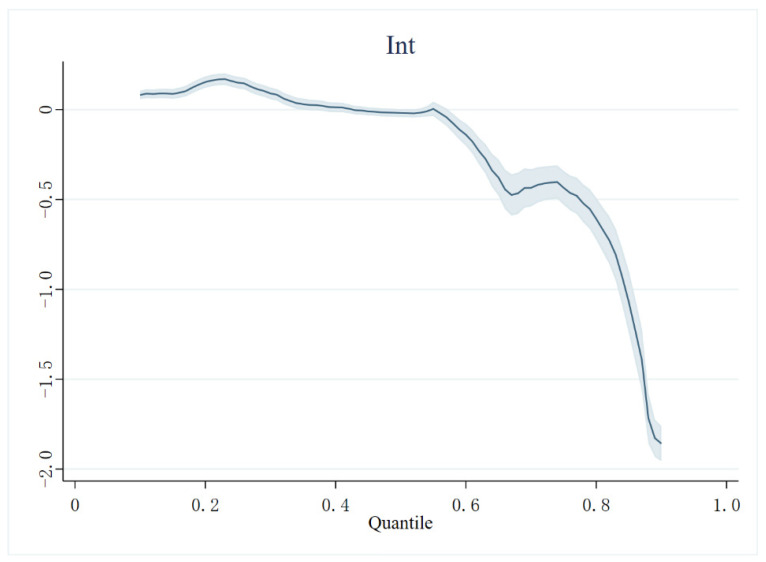
Quantile regression results.

Since we found differences in the marginal effects exhibited by the Internet at different levels of health in the grouped regressions, we wanted to further analyze the variation in the marginal effects of the Internet at different levels of health deprivation. We chose to use a quantile regression model for this part of the empirical evidence because the quantile model focuses more on the conditional distribution. The model can reflect the full picture of the entire conditional distribution by estimating different conditional quartiles. Moreover, the objective function of quantile regression is a weighted average of the absolute values of the residuals, which is less affected by extreme values than the method of minimizing the sum of squares of the residuals. We calculated the coefficients at different quartiles to an accuracy of 0.01 and plotted them in Figure 2 with 95% confidence intervals.

The quantile regression results in Figure 2 show that the negative effect of Int on HI changes from positive to negative and increases gradually as the quantile increases. This suggests that Internet use exacerbates HI in individuals with low levels of health deprivation, which corresponds to the models (16) and (17) above. At quartiles below 0.5, the marginal effect of Int slowly decreases to 0. This suggests that the negative effect of the Internet is greater among individuals with lower levels of health deprivation, but as the level of health deprivation increases, the positive effect of the Internet gradually overshadows the negative effect. After the quantile exceeds 0.5, the marginal effect of Int crosses downward to 0, with a negative effect that declines rapidly. This suggests that Int can significantly reduce HI for those with higher levels of health deprivation, and that this effect also rises rapidly as deprivation deepens. Once the quantile exceeds 0.8, the marginal effect of Int rises rapidly again, a result that implies that the strongest effect of Int can be found in people with deep health deprivation. In summary, for the healthier population, using the Internet increases their HI, so their use of the Internet may not be a good choice from a health perspective. However, compared to healthy people, people with deep health deprivation need the Internet more, and the Internet can be very effective for such people.

#### 4.5.3. Internet Access Opportunities Differences

**Figure 3:**
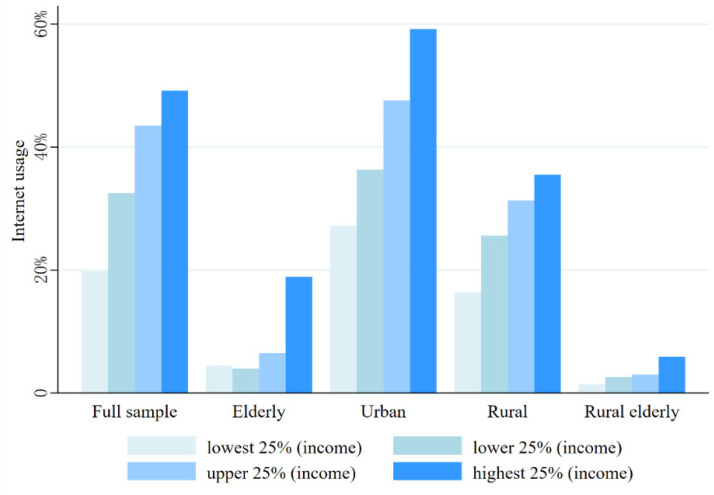
Internet usage by different groups.

From the above analysis, we find that the Internet can play a large role in the reduction of HI, which seems to indicate that the Internet can bring a “dividend”. However, we consider that the Internet requires the support of digital devices, such as computers and cell phones, which means that access to the Internet is related to household income. Therefore, we want to examine the differences in Internet accessibility among different groups. We first ranked individuals according to their annual household income. Then, we divided the ranked individuals into four equal parts to obtain four segments with household income in the lowest 25%, lower middle 25%, upper middle 25%, and highest 25%. We calculated the Internet usage rate, i.e., the proportion of the number of individuals using the Internet to the total number of individuals, in each of the four partial groups, and the calculation results are shown in Figure 3. Using this method, we calculated the rates of Internet use for the elderly sample, the urban sample, the rural sample, and the rural elderly sample, as well as the full sample. The results are shown in Figure 3.

We found a huge difference in Internet usage rates between higher-income households and lower-income households. In the full sample, the Internet usage rate of the top 25% group was 49.2%, while the bottom 25% was only 19.8%, and the Internet usage rate of the top 25% was 2.477 times higher than that of the bottom 25%. This means that although Internet use contributes significantly to health inequalities, low-income groups do not enjoy the “digital dividend” equitably, creating a “digital divide”. In the older age group, Internet usage is significantly lower for all income levels than for the full sample. Moreover, the difference in Internet usage between the top 25% and the bottom 25% is even greater, with the Internet usage rate of the top 25% being more than four times that of the bottom 25%, much higher than the corresponding value of the full sample. Thus, the elderly and the elderly poor face a greater “divide” than the younger group. Then, comparing the Internet usage rates of urban and rural groups, we find that the Internet usage rates of rural groups are lower than those of urban groups in all income brackets. The relative difference in Internet usage between the poorest and richest groups in rural areas is not much different from that in urban areas. This suggests that Internet penetration in rural areas still needs to be improved and that rural residents are at a greater disadvantage in terms of Internet access. Finally, we find that Internet usage is lower among the rural elderly, and the relative gap between the poorest and richest groups is also quite large, making them the least affected by the “dividend”.

In summary, we can determine that while the Internet has played a positive role as a “dividend”, it has also been accompanied by a huge “divide”, meaning that the Internet is a double-edged sword. We hope that, in addition to the positive effects of the Internet, the lack of access to disadvantaged groups will be noticed. This does not mean that we should be disappointed with the construction and development of the Internet; on the contrary, it means that the development of the Internet should be more comprehensive, so that more groups can enjoy the “dividends” of the Internet.

## 5. Discussion

Currently, despite the increasing improvement in China’s health care conditions, the rising living standards of the population, and the increasing importance people attach to their health, people’s health levels are still heterogeneous. In our sample, residents’ health is divided into five levels, and the average value of health deprivation is higher than 2, which indicates that health inequality will become a key concern in the longer term. Meanwhile, Internet technology is developing at a rapid pace, offering new ideas for building China’s future. The Internet not only brings quality and convenient online services to residents, but also causes changes in lifestyles and habits. Therefore, the first step of our study is to see if the Internet has created a “dividend” or a “divide” for the population, with the aim of providing empirical evidence for the spread of the Internet.

With these considerations in mind, we used CFPS data from 2014 to 2018 to measure health inequality in China based on resident health self-assessment to investigate whether Internet use can improve health inequality. We determined the effect of the Internet on health inequality through a two-way fixed effects model. The findings suggest that the Internet can reduce health inequalities. We conducted variable substitution tests, endogeneity tests, selection bias tests, and functional form-setting bias tests for this finding, which ultimately proved our findings to be robust. In addition, we searched for three channels through which interconnection affects health inequality. First, the Internet reduces the income inequality of the population, which improves the living conditions of the population as well as health care accessibility, and therefore reduces health inequality. Second, the Internet reduces depression among the population, which makes it easier for people with poor health to obtain psychological comfort, which helps them to be prevented and recover from mental illness. Finally, the Internet has facilitated the dissemination of health knowledge among the population. Although indulgence on the Internet may reduce the frequency of physical activity, the positive effects of Internet information dissemination are greater. For example, the popularity of fitness apps has increased health consciousness among the population, which is reflected in the increased frequency of physical activity brought about by the Internet.

We find that the impact of the Internet on health inequalities is heterogeneous, with the Internet having a stronger marginal effect on unhealthy people, older people, rural groups, and women. In general, they are more likely to be socially disadvantaged, which allows Internet use to significantly increase their probability of accessing help. This help may be reflected not only in life support, but also in psychological comfort, both of which would help them reduce their level of health deprivation and health inequalities. We also focused on the differences in the role of the Internet across levels of health deprivation. Our findings favor a greater role for the Internet among disadvantaged groups, reflecting the stronger marginal effect of the Internet and greater “dividends” among those with higher levels of health deprivation. In contrast, we find that disadvantaged groups are disadvantaged in terms of Internet access, especially rural, elderly, and low-income groups, which means that while we focus on the “dividend”, we cannot forget the “divide” created by Internet development.

Our study still has limitations. Part of this is due to data limitations; due to changes in the questionnaire, we only used data from 2014 onwards, which may have led to some measurement error. In addition, the health self-assessment we used only represents the health level at the time of the visit, which may cause the actual health level to be inconsistent with the questionnaire if the respondent suffers an acute medical shock in a short period of time. Moreover, the questionnaire data we used may have some survey bias. In short, we tried as hard as we could to make sure the model was not biased, and we came to the conclusion that Internet use in general can help reduce health inequality.

## 6. Conclusions

Based on CFPS data from 2014 to 2018, we empirically examined the impact of Internet use on health inequality among Chinese residents, examining the roles of income inequality, depressed mood, and health consciousness in this context, as well as the heterogeneous effect of the Internet on different groups. We found that: (1) Internet use can reduce health inequality among Chinese residents. (2) The Internet can reduce residents’ income inequality, reduce depression, increase the frequency of physical activity participation, and thus reducing health inequality. (3) The marginal effect of the Internet was higher in the unhealthy group, the elderly group, the urban group, and the female group. As the level of health deprivation increases, the Internet’s effect on health inequality mitigation becomes stronger. (4) There are significant differences in Internet access among Chinese residents in different income brackets, with poorer Internet access among the low-income, elderly, and rural groups.

Based on the above findings, we believe that the following recommendations should be adopted to help Chinese residents improve their health and reduce health inequality: First, we recommend accelerating the construction of universal Internet infrastructure and the introduction of digital devices into rural areas. We should focus on regional and urban–rural disparities in Internet development, reduce the difficulty of Internet access, and reduce the Internet access “divide”. Second, we should accelerate the reform of Internet hardware and software to help the elderly become engaged in Internet use. Relevant departments should lower the threshold of Internet access for the elderly, especially the Internet penetration among rural elderly groups, focus on the construction of Internet communities for the elderly and help them to facilitate their cultural exchange and life online. Lastly, government departments should improve how the Internet is regulated and how it works. We should also try to make people less addicted to the Internet, keep it from taking up too much of their free time, make it less harmful to healthy groups, and make the most of the Internet’s “dividend”.

## Figures and Tables

**Table 1:** Descriptive statistics.

Variables Type	Variable	Variable Description	Full Sample		Internet (Yes)	Internet (No)	Diff
			Mean	Sd	Mean	Mean	Mean
Explained variable	HI	Calculated according to Formula (2)	2.217	2.052	1.620	2.540	−0.920 ***
Explanatory variable	Int	1 = yes, 0 = no	0.351	0.477			
Mediating variables	Ini	Income inequality level	0.394	0.105	0.383	0.401	−0.018 ***
	Dep	CES-D scale score	3.902	3.432	3.564	4.084	−0.520 ***
	Exe	Number of physical exercises per week	2.310	3.197	2.421	2.251	0.170 ***
Control variables	Age	Resident age	49.058	15.230	37.581	55.256	−17.675 ***
	Urb	1 = yes, 0 = no	0.472	0.499	0.619	0.393	0.225 ***
	Gen	1 = male, 0 = female	0.483	0.500	0.507	0.470	0.037 ***
	Mar	1 = married, 0 = unmarried	0.843	0.363	0.765	0.886	−0.120 ***
	Reg	1 = agricultural household registration, 0 = else	0.719	0.450	0.587	0.790	−0.203 ***
	Pin	The logarithm of per capita household net income	8.865	1.734	9.325	8.616	0.708 ***
	Fam	Number of family members	4.212	1.963	4.242	4.195	0.046 **
	GDP	Logarithm of GDP per capita	10.732	0.418	10.802	10.694	0.108 ***
	Urr	Urban population ratio	0.575	0.123	0.596	0.565	0.031 ***
	Gov	Financial revenue/GDP	0.109	0.030	0.111	0.109	0.002 ***
	Fin	Logarithm of fiscal expenditure	8.607	0.411	8.646	8.586	0.060 ***
	Med	Number of medical institutions/Permanent population	7.627	2.606	7.385	7.758	−0.372 ***

Note: *** and ** represent the significance levels of 1% and 5%.

**Table 2:** Benchmark regression results.

Variables	(1)	(2)	(3)	(4)	(5)
Int	−0.920 ***	−0.193 ***	−0.185 ***	−0.142 ***	−0.187 ***
	(−49.646)	(−8.499)	(−8.095)	(−6.092)	(−7.803)
Age		0.038 ***	0.037 ***	0.038 ***	0.037 ***
		(54.993)	(51.789)	(52.424)	(49.785)
Urb		−0.078 ***	−0.076 ***	−0.079 ***	−0.074 ***
		(−3.949)	(−3.807)	(−3.943)	(−3.631)
Gen		−0.448 ***	−0.448 ***	−0.454 ***	−0.457 ***
		(−26.059)	(−26.129)	(−26.489)	(−26.772)
Mar		−0.037	−0.006	−0.001	0.020
		(−1.522)	(−0.264)	(−0.034)	(0.816)
Reg		0.098 ***	0.085 ***	0.101 ***	0.084 ***
		(4.398)	(3.761)	(4.384)	(3.566)
Pin			−0.042 ***	−0.043 ***	−0.037 ***
			(−8.243)	(−8.400)	(−7.210)
Fam			−0.025 ***	−0.027 ***	−0.037 ***
			(−5.493)	(−5.793)	(−7.736)
GDP				−0.057	−0.434
				(−0.888)	(−1.454)
Urr				−0.731 ***	0.010
				(−3.550)	(0.011)
Gov				0.183	1.436
				(0.391)	(0.707)
Fin				−0.145 ***	0.141
				(−4.264)	(0.420)
Med				−0.058 ***	−0.008
				(−11.518)	(−0.148)
Provincial-fixed effect					YES
Year-fixed effect					YES
Constant	2.540 ***	0.619 ***	1.143 ***	3.781 ***	4.477
	(231.393)	(12.136)	(15.371)	(7.269)	(1.542)
Observations	51,375	51,375	51,375	51,375	51,375
R-squared	0.046	0.108	0.110	0.112	0.120

Note: The brackets represent t values, with *** representing the significance levels of 1%.

**Table 3:** Robustness test results.

Variables	(6)	(7)	(8)	(9)
	Change Variable	IV	TEM	PSM
Internet time	−0.002 **			
	(−2.408)			
Int		−0.975 ***	−0.922 ***	−0.920 ***
		(−4.690)	(−9.131)	(−49.646)
Control variables	YES	YES	YES	YES
Constant	3.899	7.559 **	5.025 *	
	(1.286)	(2.391)	(1.734)	
Observations	51,375	51,375	51,375	51,375
R-squared	0.119	0.101		

Note: The brackets represent t values, with ***, **, and * representing the significance levels of 1%, 5%, and 10%.

**Table 4:** Mechanism test results.

Variables	(10)	(11)	(12)	(13)	(14)	(15)
	Ini	HI	Dep	HI	Exe	HI
Int	−0.020 ***	−0.173 ***	−0.078 **	−0.173 ***	0.599 ***	−0.170 ***
	(−16.839)	(−7.210)	(−2.080)	(−7.565)	(16.233)	(−7.087)
Ini		0.698 ***				
		(7.770)				
Dep				0.168 ***		
				(66.732)		
Exe						−0.028 ***
						(−10.136)
Control variables	YES	YES	YES	YES	YES	YES
Provincial-fixed effect	YES	YES	YES	YES	YES	YES
Year-fixed effect	YES	YES	YES	YES	YES	YES
Constant	2.363 ***	2.827	13.352 **	2.233	16.497 ***	4.942 *
	(16.577)	(0.971)	(2.513)	(0.802)	(3.549)	(1.703)
Observations	51,375	51,375	51,375	51,375	51,375	51,375
R-squared	0.508	0.121	0.110	0.190	0.089	0.122

Note: The brackets represent t values, with ***, **, and * representing the significance levels of 1%, 5%, and 10%.

## Data Availability

Not applicable.
